# Malaria parasitemia and its association with CD4 cells, viral load and haematological parameters among HIV-infected children < 15 years in the Bonasssama Health District, Douala, Cameroon: Prevalence and risk factors

**DOI:** 10.1016/j.parepi.2024.e00390

**Published:** 2024-11-01

**Authors:** Ambe Fabrice Ngwa, Ekwi Damian Nsongmayi, Tanyi Pride Bobga, Bih Vanessa Tita, Judith Ngong Nyeme, Nyanjoh Eugine Mbuh

**Affiliations:** a Department of Microbiology and Parasitology, Faculty of Science, University of Buea, Cameroon; b Department of Medical Laboratory Sciences, Faculty of Health Sciences, University of Buea, Cameroon; c School of Medical and Biomedical Sciences, Fomic Polytechnic University, Buea, Cameroon; d International School for Nurses and Technico-Sanitary Personnels, Douala, Cameroon; e Department of Biomedical and Medico Sanitary Sciences, Faculty of Science, University of Ebolowa, Cameroon; f Department of Nursing, Faculty of Health Sciences, University of Buea, Cameroon

**Keywords:** Cameroon, CD4 cells, Haematological parameters, HIV, Malaria, Viral load

## Abstract

**Background:**

One of the major causes of morbidity and death in children is malaria, and HIV infection and other factors may make the situation worse. This study aimed to assess the prevalence of malaria parasitemia among HIV-infected children under 15 years in the Bonassama Health District, Douala, Cameroon, and investigate its association with CD4 cell counts, viral load, and haematological parameters.

**Methods:**

The study was a cross-sectional study involving 287 HIV-infected children <15 years and convenient sampling was used to enrol participants. A semi-structured questionnaire was used to obtain the characteristics of the participants from the caregivers. Venous blood was collected; blood films were made and stained using Giemsa for parasite detection. Full blood count, CD4 level and viral load were measured using a haematology auto-analyzer, pima counter and genexpert, respectively. Data were analysed using SPSS, and the chi-square test was used to assess the association. Predisposing factors to malaria were evaluated using multivariable logistic regression, and a *p* < 0.05 was considered significant.

**Results:**

The overall prevalence of malaria and anaemia was 31.01 % and 25.44 %, respectively. Malaria prevalence was significantly higher in children <5 years (42.68 %, *p* < 0.001), those presented with fever (40.70 %, *p* = 0.047), children not on antiretroviral therapy (ART) (28.6 %, *p* = 0.02) and cotrimoxazole (28.6 %, p = 0.02). Children <5 years (AOR = 1.81, 95 % 1.19–2.75), those between 5 and 9 years (AOR = 1.61, 95 % CI 1.11–2.48), children not on ART(AOR = 2.2, 95 % 1.03–4.74) and Cotrimoxazole (AOR = 9.08, 95 % 2.33–43.46), febrile children (AOR = 1.72, 95 % 1.01–2.11), children with viral load >3000 copies/μL(AOR = 2.933, 95 % 1.36–6.49), and CD4 count <200cells/ μL (AOR = 3.09, 95 % 2.08–4.6) were factors associated with malaria parasitemia among HIV-infected children. Haemoglobin levels (*p* = 0.0016), White Blood Cells (*p* = 0.002), Red Blood Cells (*P* < 0.001), neutrophils count (*p* < 0.001), and platelet counts (*p* = 0.0164) were significantly lowered among malaria/HIV children compared to HIV-infected children.

**Conclusion:**

The study concludes that HIV-infected children under 5 years, especially those not on ART or cotrimoxazole, are at a significantly higher risk for malaria and related haematological issues. This underscores the necessity for targeted malaria screening and treatment in this vulnerable group. Public health strategies should prioritize enhancing access to ART and cotrimoxazole to mitigate these risks and improve overall health outcomes.

## 1 Introduction

Malaria and HIV/AIDS co-infection pose a significant health challenge, particularly among children under 15 years old in Sub-Saharan Africa, where both diseases are endemic (Opeyemi and Obeagu, 2023). Malaria, caused by Plasmodium parasites transmitted through the bite of infected Anopheles mosquitoes, is a leading cause of morbidity and mortality in this age group. In 2022, WHO reported 249 million cases of malaria globally and an estimated 608,000 deaths, approximately 94 % of the malaria cases and deaths were in the WHO African Region and children accounted for about 80 % of all malaria deaths in the World Health Organization (WHO) African region. In Cameroon, Malaria is the most widespread endemic disease and is responsible for more than 2 million reported cases annually, and the prevalence of malaria among children was 24 % (Ndoula et al., 2024; Ojongnkpot et al., 2023). HIV compromises the immune system by targeting and destroying CD4 T cells, which are crucial for coordinating the body's immune response. As the number of these cells declines, the body becomes less capable of fighting off infections. This weakened immune state significantly increases the risk of opportunistic infections, such as tuberculosis, pneumonia, and malaria (Mirzohreh et al., 2022). HIV is a well-established global health burden, with 39 million cases, 1.5 million HIV-infected children <15 years and 630,000 deaths in 2022, and Sub-Saharan Africa has the lion's share of the global HIV burden (approximately 70 %)(Moyo et al., 2023; Stover and Glaubius, 2024).

Several epidemiological studies conducted in sub-Saharan Africa have investigated the prevalence of malaria among HIV/AIDS patients under 15 years old (Mirzohreh et al., 2022). Approximately 70 % of the world's HIV-infected population lives in Sub-Saharan Africa including Cameroon, where 350 million people are exposed to malaria and it is the third-highest cause of HIV-related morbidity (Kwenti, 2018; Obase et al., 2023). Malaria and HIV/AIDS co-infection in children under 15 years old can have severe health consequences, including increased risk of severe malaria, anaemia, respiratory distress, cerebral malaria, coma, multiorgan failure, cognitive impairment, and mortality (Jacques et al., 2020). Children are generally susceptible and vulnerable to malaria (Kwenti, 2018). With HIV co-infection they are increasingly vulnerable to malaria-related complications because HIV-induced downregulation of CD4+ T cells and decreases in CD8+ T-cell counts. Upregulation of parasitemia, and also malaria has been associated with increased HIV replication in co-infected individuals (Chukwuocha et al., 2019). Malaria and HIV/AIDS co-infection has a negative or positive impact on the efficacy of antimalarial and/or ARV drugs in patients concomitantly receiving treatment for malaria and ARV for HIV infection (Kwenti, 2018; Mirzohreh et al., 2022). The coinfection of malaria and HIV/AIDS can negatively impact the efficacy of both antimalarial and ARV drugs due to complex drug interactions and altered pharmacokinetics (Kwenti, 2018; Obeagu and Obeagu, 2024). HIV medications might affect the metabolism of antimalarials, leading to reduced drug effectiveness or increased toxicity. Additionally, the immunosuppressive effects of HIV and the increased disease burden from severe malaria can further complicate treatment outcomes (Kwenti, 2018; Obeagu and Obeagu, 2024). Conversely, certain antimalarial drugs can alter the pharmacokinetics of ARVs, leading to reduced efficacy or increased toxicity. This interplay underscores the need for careful monitoring and potential dose adjustments to optimize treatment outcomes and minimize complications(Kwenti, 2018).

Both malaria and HIV account for over 2 million deaths globally every year, and the average prevalence of malaria among HIV-positive children was 39.4% (Mirzohreh et al., 2022). The prevalence of malaria/HIV co-infection in Cameroon varies from one region to the other; ranging from 2.24 % in Bamenda (Njunda et al., 2012), to 29.5 % in Douala (Nkuo-Akenji et al., 2011) and 24.8 % among HIV infected children in Mutengene (Bate et al., 2016). Although there have been some reports of malaria in HIV patients in Cameroon (Bate et al., 2016; Nkuo-Akenji et al., 2011; Sandie et al., 2019), no study has specifically focused on prevalence and factors associated with malaria among HIV-infected children in Douala, Cameroon. Understanding the prevalence of malaria among HIV/AIDS patients under 15 years old in Douala, Cameroon is critical for informing public health policies, optimising treatment protocols, and reducing the burden of both diseases in this vulnerable population. Thus, there is a need to assess Malaria parasitemia and its association with CD4 cells, viral load and haematological parameters among HIV-infected children <15 years in the Bonasssama Health District, Cameroon.

## 2 Materials and methods

### 2.1 Study design and population

A hospital cross-sectional study was carried out among HIV-infected children aged <15 years who came to the HIV/AIDS treatment center of the Bonassama District Hospital. This study was carried out from October 2023 to April 2024. Douala is a cosmopolitan town with a population of over two million inhabitants, and with several mosquito breeding sites that persisted all year long as well as a high prevalence of mosquito resistance to DDT and permethrin insecticides (Antonio-Nkondjio et al., 2012) .

### 2.2 Inclusion criteria

All HIV-infected children aged <15 years who visited the HIV/AIDS treatment center of the Bonassama District Hospital for treatment and whose parent/legal guardian completed the assent form were included in the study. Also, those who were feverish were recruited in the study.

### 2.3 Exclusion criteria

All the children recently diagnosed with malaria and on antimalarial in the past two weeks were excluded from the study.

### 2.4 Ethical considerations

Ethical review and clearance were sought from the Institutional Review Board (IRB/FHS UB) with an authorization N° 2024/0001/UB/HOD/MLS/FHS, and administrative clearance from the Regional Delegation of Public Health, and the director of the Bonassama District Hospital. Before enrolment, a detailed explanation of the study and its potential benefits was given to parents/legal guardians of the children who were then invited to participate in the study.

### 2.5 Sample size

The sample size was estimated using the Lorenz formula; *n* = *z*^2^*p*(1-*p*)/*d*^2^, where *z* = *Z* score for 95 % confidence interval = 1.96, *p* = past prevalence, and *d* = acceptable error (5 %). We used the prevalence of Malaria infection and anaemia in HIV- infected children in Mutengene, Southwest.

Cameroon, of 24.8 % (Bate et al., 2016). The sample size attained was 287 participants.

### 2.6 Sampling technique

A convenient sampling technique was used to recruit the study participants.

### 2.7 Data collection

#### 2.7.1 Administration of questionnaires

A structured questionnaire was pretested and reviewed by an expert to ensure validity and reliability. The pretested data were entered into SPSS and reliability calculated, and Cronbach's alpha calculated value was 0.82 for the questions based on the use of malaria preventive measures (long-lasting insecticide-treated nets (LLIN), insecticide residual spraying (IRS), presence/absence of stagnant water or bushes around the home), use of and duration on ART, the type of ART and the utilization of cotrimoxazole . There questionnaires were then administered to each parent/guardian and interviews were done in English and French, and pidgin English where necessary. The questionnaire was translated by three different public health experts with a background in language translation and interpretations and harmonized to ensure the accuracy of the translated version. The questionnaire sought information on the sociodemographic characteristics (age, gender, occupation of guardian/parent and residence), use of malaria preventive measures (long-lasting insecticide-treated nets (LLIN), insecticide residual spraying (IRS), presence/absence of stagnant water or bushes around the home), use of and duration on ART, the type of ART and the utilization of cotrimoxazole.

#### 2.7.2 Sample collection and laboratory diagnosis

##### 2.7.2.1 Sample collection

Venous blood was collected into well-labelled Ethylene Diamine Tetra Acetate (EDTA) tubes, and thick and thin blood films were made for the detection and speciation of malaria parasites respectively according to the method described by Cheesbrough (2005).

##### 2.7.2.2 Detection of malaria parasitemia

After fixing air-dried thin blood films with absolute methanol, both thick and thin blood films were stained with 10 % Giemsa for 30 min and examined under the ×100 (oil immersion) objective of an Olympus® BX 40 F light microscope (Olympus Optical Co. Ltd., Japan), for the detection and identification of malaria parasites, respectively, using the bench aids of Cheesbrough (Cheesbrough, 2005). Slides were declared negative if no parasites were found after examining 100 high-power fields. Slides were read by two independent parasitologists, and in the case of any disparity, they were read again by a third person. The amount of parasitemia was calculated for each positive smear by comparing the number of parasites to 200 leucocytes and assuming an 8000 leucocyte count (Cheesbrough, 2005). Parasitaemia was categorized as low (< 1000 parasites/μL blood), moderate (1000–4999 parasites/μL blood), high (5000–99,999 parasites/μL blood), and hyperparasitemia (≥100,000 μL).

##### 2.7.2.3 Determination of CD4 cell and viral load

The blood specimen was used for determination of CD4 cell count using flow cytometry (pima counter) and the counts were categorized according to the standards of the WHO, as severe when counts <200 cells/μl; low (200–349 cells/μl); moderate(350–499 cells/μl) and high when counts ≥500 cells/μl (Organization, W. H, 2009). Viral load was measured using Genexpert according to the manufacturer's instructions.

##### 2.7.2.4 Haematology

Following the manufacturer's instructions, the URIT-3300 automated haematology analyzer (URIT Medical Electronic CO., LTD. Jiuhua Road, Gungxi, China) was used to determine full blood count, white blood cell (WBC),red blood cell (RBC) and platelet counts, haemoglobin concentration (Hb), haematocrit (Hct), mean corpuscular volume (MCV), mean corpuscular haemoglobin (MCH), mean corpuscular haemoglobin concentration (MCHC) and platelets were measured. Anaemia was defined as Hb < 11.0 g/dL and categorized as mild (>10.0 g/dL and < 11 g/dL), moderate (Hb between 7.0 g/dL and 10.0 g/dL) and severe (Hb <7 g/dL) as described by Cheesbrough (2005).

##### 2.7.2.5 Data analyses

Data were recorded on register forms and entered in a Microsoft Excel database in a secure computer and analysis was done with SPSS version 20 and EPI info version 7. Data were statistically described in terms of frequencies and percentages. The significance of the difference in prevalence with respect to sociodemographic factors were explored using Pearson's chi-square test. A *p*-value of less than 0.05 was considered statistically significant. Multivariate analysis was applied to analyze risk factors associated with malaria among HIV-infected children.

## 3 Results

### 3.1 Characteristics of the study participants

Two hundred and eighty-seven (287) HIV-infected children aged between 1 and 14 years were involved in this study. The mean age of the participants was 6.5 (SD = 1.19), and the median age was 7 years. The study population comprised 168 (58.54 %) females and 119 (41.46 %) males in 3 different age groups. One hundred and twenty-six (43.90 %) of the participants fall within the age group 5–9 years, and the age group <5 years constituted 82 (28.57 %) participants. Ninety-six (33.45 %) of the participants have a CD4 count of <500 cells/μL. A greater proportion of the parents/guardians of the children have attained the secondary level of education (*n* = 125; 43.55 %), followed by those having primary school education (*n* = 83;

28.92 %) and University (*n* = 79; 27.53 %) (Table 1).

**Table 1:** Characteristics of the study participants.

Variables	Frequency	Percentage
Age/year	6.50(SD = 1.19)	
10–14	79	27.53
5–9	126	43.90
<5	82	28.57
Gender		
Male	119	41.46
Female	168	58.54
Fever		
No	201	70.03
Yes	86	29.97
CD4 T cell/μl		
≥ 500	96	33.45
350–499	78	27.18
200–349	45	15.68
< 200	68	23.69
ART USAGE		
Yes	244	85.02
No	43	14.98
Type of ART		
PI	58	27.18
NRI	91	37.63
NNRI	95	35.19
Cotrimoxazole usage		
Yes	97	33.80
No	190	66.20
LLIN Usage		
Yes	79	27.53
No	208	72.47
Level of education of guardian/parents		
University	79	27.53
Secondary	125	43.55
Primary	83	28.92
Anaemia status		
Negative	207	72.13
Positive	80	27.87
Viral load copies/mL		
<3000	118	41.11
>3000	169	58.89

### 3.2 Prevalence of malaria and Anaemia among HIV-infected children

**Fig. 1:**
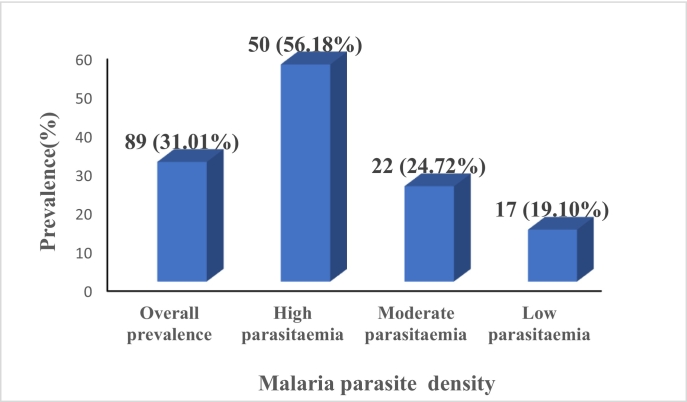
The overall prevalence of malaria parasite and density among HIV-infected child.

Out of the 287 HIV-infected children aged between 1 and 14 years screened for malaria, 89 of our study population were positive for malaria parasite, giving a prevalence of 31.01 % as shown in Fig. 1. Most (*n* = 50; 56.18 %) of the positive cases have parasitemia levels between 5000 and 99,999 parasites/μL blood (high), followed by (*n* = 22; 24.72 %) with 1000–4999 parasites/μL blood (moderate) and (n=17; 19.10 %,) < 1000 parasites/μL blood (low).

**Fig. 2:**
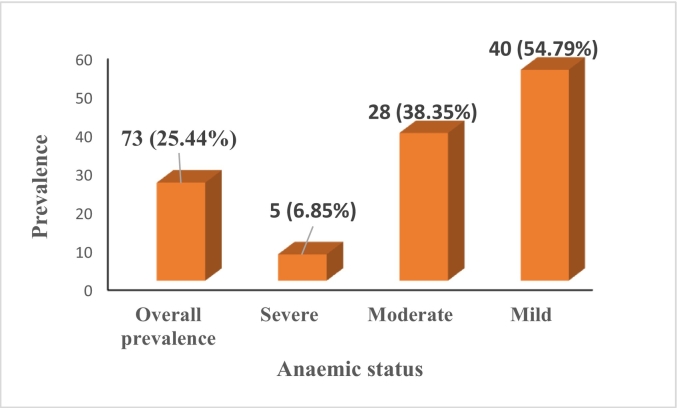
Prevalence of anaemia among HIV- infected children.

The overall anaemia prevalence in HIV-infected children was 25.44 % (73). Of the 73 anaemic cases most had mild anaemia (*n* = 40; 54.79 %), followed by moderate (*n* = 28; 38.35 %), and. Severe (n = 5; 6.85 %) (Fig. 2).

### 3.3 Prevalence of malaria in the study population with regards to gender, fever, use of ART, CD4 T cell counts, viral load, type of ART and use of cotrimoxazole

Malaria prevalence was significantly higher in children <5 years (*n* = 35, 42.68 %) compared to those between 5 and 9 years (*n* = 34; 26.98 %) and 10 to 14 years (*n* = 20; 25.32 %) (χ2 = 38.34, *p* < 0.001). Within both categories of patients, febrile patients had a higher prevalence of malaria (n = 35; 40.70 %) than their afebrile counterparts (*n* = 54; 26.87 %) and there was a significant association between fever and malaria parasitemia (χ2 = 5.78, *p* = 0.047). Malaria parasite prevalence though not significant was slightly higher among females (*n* = 53; 31.55 %) than males (*n* = 36; 30.25 %) (χ2 = 0.21, *p* = 0.90) (Table 2).

**Table 2:** Prevalence of malaria in the study population with regards to gender fever use of ART CD4 T cell counts viral load type of ART and use of cotrimoxazole.

Variables	Number examined	Malaria parasitemia n (%)	Chi-square (X^2^)	P- value
Age/year				
10–14	79	20(25.32)	38.34	< 0.001
5–9	126	34(26.98)		
<5	82	35(42.68)		
Gender				
Male	119	36(30.25)	0.21	0.90
Female	168	53 (31.55)		
Fever				
No	201	54 (26.87)	5.78	0.047
Yes	86	35(40.70)		
CD4 T cell/μL				
≥ 500	96	20(20.83)	10.18	0.005
350–499	78	19(24.36)		
200–349	45	17(37.78)		
< 200	68	33(48.53)		
ART usage				
Yes	244	66(27.05)	6.13	0.029
No	43	23(53.49)		
Type of ART				
PI	58	5(8.62)	7.03	0.022
NRI	91	32(35.16)		
NNRI	95	38(40.00)		
Cotrimoxazole usage				
Yes	97	15(15.46)	7.537	0.02
No	190	74(38.95)		
LLIN Usage				
Yes	79	21(26.58)	2.78	0.11
No	208	68(32.69)		
Level of education of guardian/parents				
University	79	21(26.58)	1.08	0.57
Secondary	125	38(30.40)		
Primary	83	30(36.14)		
Anaemia status				
Negative	214	53(25.60)	0.05	0.99
Positive	73	36(49.32)		
Viral load copies/mL				
<3000	118	32(27.19)	33.4	< 0.001
>3000	169	57(33.73)		

The highest malaria parasite prevalence was observed in children with CD4 T cell count <200 cells/μl (*n* = 33; 48.53 %), followed by those with CD4 T cell count between 200 and 349 (*n* = 17; 37.78 %). In comparison, the lowest was recorded in children with CD4 T cell count ≥500 cells/μL (n = 20; 20.83 %,) and there was a significant association (χ2 = 10.18, *p* = 0.005). The prevalence of malaria in children who were not on ART (*n* = 23; 53.49 %,) was higher than in children who were on ART (*n* = 66; 27.05 %,) and there was a significant association between ART and malaria parasitemia (χ2 = 6.13, *p* = 0.029). There was a statistically significant difference between the type of ART and malaria prevalence and the highest prevalence was observed among children on NNRTI (*n* = 38; 40.00 %,), while the lowest prevalence was recorded among children on PI (n = 5; 8.62 %) (χ2 = 7.03, *p* = 0.022) (Table 2).

As shown in Table 2, children on cotrimoxazole (*n* = 15; 15.46 %) had significantly (χ2 = 7.537, p = 0.02) lower malaria parasite prevalence compared to those not on cotrimoxazole (38.95 %, *n* = 74). Viral load was statistically associated with malaria parasitemia and a higher prevalence was obtained among children with viral loads >3000 copies/mL (*n* = 62; 36.69 %). In the study participants, children not using insecticide-treated nets (ITNs) had a higher prevalence of malaria parasitemia (*n* = 78; 37.50 %) than those who used ITNs (*n* = 21; 26.58 %), but the difference was not significant (χ2 = 2.78, *p* = 0.11).

### 3.4 Factors associated with malaria among HIV-infected children <15 years

In bivariate logistic regression children <5 years (AOR = 1.47, 95 % CI 1.06–2.17, *p* = 0.007), and those between 5 and 9 years (AOR = 1.88, 95 % CI 1.24–2.84, *p* = 0.002), children not on ART(AOR = 1.78, 95 % CI 1.38–3.56, *p* = 0.018), suffering from fever (AOR = 1.24, 95 % CI 1.01–2.06, *p* = 0.04), viral load >3000 copies/μL (AOR = 4.32, 95 % CI 2.32–8.02, *p* = 0.0002), females, children not on Cotrimoxazole (AOR = 2.1, 95 % CI 1.73–4.74, *p* = 0.044), NNRTI (AOR = 4.09, 95 % CI 1.28–14.4, *p* = 0.023) and CD4 count <200 cells/ μL(AOR = 3.22, 95 % CI 2.18–4.75, < 0.001) were significantly associated to malaria parasitemia among HIV infected children while the remaining variables were not observed to have any significant association with malaria.

In multivariable logistic regression analysis, children in the age groups <5 years and between 5 and 9 years were significantly associated with 1.81 and 1.61 higher odds of infection, respectively. In addition, females (AOR = 3.08, 95 % CI 1.37–6.93, p = 0.007) were three times more likely to be significantly associated with Plasmodium infection, the odds of malaria infection were 3 times higher in study participants with CD4+ T cell count <200 cells/μL as compared to those who have CD4+ T cell count >500 cells/μL (AOR = 3.09, 95 % CI 2.08–4.6, *p* < 0.001), and the odds of malaria was significantly two times higher in febrile children compared to afebrile children (AOR = 1.72, 95 % CI 1.01–2.11, *p* = 0.03). Children not on ART (AOR = 2.2, 95 % CI 1.03–4.74, *p* = 0.045) and cotrimoxazole (AOR = 9.08, 95 % CI 2.33–43.46, *p* = 0.0017) were two and nine times, respectively, more likely to be suffering from malaria. Also, Children with a viral load >3000 copies/mL were 3 times more likely to be infected with plasmodium than those having viral load <3000 copies/mL (AOR = 3.13, 95 % CI = 1.36–6.49, p = 0.007), and children on NNRTI were two times positivity associated to malaria infection (AOR = 2.04, 95 % CI = 3.10–5.82, *p* = 0.035) (Table 3).

**Table 3:** Factors associated with malaria among HIV-infected children <15 years.

Variables	COR (95 % CI)	P- value	AOR (95 % CI)	P- value
Age/year				
10–14	1		1	
5–9	1.47 (1.06–2.17)	0.007	1.61 (1.11–2.48)	0.006
<5	1.88 (1.24–2.84)	0.002	1.81 (1.19–2.75)	0.005
Gender				
Male	1		1	
Female	2.74 (1.40–5.36)	0.004	3.08 (1.37–6.93)	0.007
Fever				
No	1		1	
Yes	1.24 (1.01–2.06)	0.04	1.72 (1.01–2.11)	0.03
CD4 T cell/μl				
≥ 500	1		1	
350–499	1.20 (0.49–2.96)	0.873	1.80 (0.55–5.85)	0.33
200–349	2.06 (0.86–4.93)	0.103	2.5 (0.8–8.2)	0.13
< 200	3.22 (2.18–4.75)	< 0.001	3.09 (2.08–4.6)	< 0.001
ART USAGE				
Yes	1		1	
No	1.78(0.88–3.56)	0.108	2.2(1.03–4.74)	0.045
Type of ART				
PI	1		1	
NRI	1.5 (0.5–2.0)	0.145	1.78(0.88–3.56)	0.108
NNRI	2.1(1.73–4.74)	0.044	2.04 (3.10–5.82)	0.035
Cotrimoxazole usage				
Yes	1		1	
No	4.09 (1.28–14.4)	0.023	9.08 (2.33–43.46)	0.0017
LLIN Usage				
Yes	1		1	
No	1.26 (0.11–1.40)	0.702	1.67 (0.87–2.47)	0.443
Level of education of guardian/parents				
University	1		1	
Secondary	1.81 (0.89–3.42)	0.077	1.52 (0.54–3.22)	0.39
Primary	1.99 (0.83–3.46)	0.145	1.82 (0.55–2.69)	0.628
Anaemia status				
Negative	1		1	
Positive	3.76 (0.23–8.06)	0.351	3(0.08–8.72)	0.456
Viral load copies/mL				
<3000	1		1	
>3000	4.32 (2.32–8.02)	0.0002	2.933 (1.36–6.49)	0.008

### 3.5 The effects of HIV/malaria coinfection on haematological parameters

The mean of haematological parameters and *P* values of the in HIV infected children with

or without malaria are shown in Table 4 below. The was a statistically significant reduction in Red blood cell count (p < 0.001), White Blood Cell count (p = 0.001), platelets count (*p* = 0.0016) and Neutophils (NEUT%) (p < 0.001) in those with malaria as compared to those without malaria. There were no significant differences between the mean values of Lymphocytes (LYM%), Monocytes (MON%), Basophils (BA%), Eosinophils (ESO%), hematocrits, Mean Cell Haemoglobin Concentration (MCHC), Mean Cell Volume (MCV) and Mean Cell Haemoglobin (MCH) in HIV-infected children with malaria, compared to those without malaria.

**Table 4:** The effects of HIV/Malaria coinfection on haematological parameters.

Haematological Parameter	HIV infected	Malaria/HIV coinfected	*t-*test	P value
WBC (×10^9^ L^−1^)	6.1 ± 0.13	5.4 ± 0.1	3.90	0.002
RBC (x10^12^ L^−1^)	4.2 ± 0.2	3.4 ± 0.3	5.442	<0.001
HB (g/dL)	11.5 ± 0.4	10.5 ± 0.2	3.64	0.0016
Haematocrit (%)	41.2 ± 0.4	29.2 ± 0.3	0.813	0.352
PLT(×10^9^ L^−1^)	139.5 ± 12.7	135.3 ± 5.3	2.91	0.0164
Lymphocytes (%)	11.7 ± 0.7	15.8 ± 0.8	0.144	0.820
Monocytes (%)	12.7 ± 0.6	10.7 ± 0.1	0.268	0.890
Basophils(%)	2.9 ± 0.2	3.2 ± 0.8	1.23	0.0781
Neutrophils (%)	58.8 ± 2.1	48.0 ± 1.4	6.314	<0.001
Eosinophils (%)	2.4 ± 0.1	6.4 ± 0.3	0.203	0.962
MCV (fL)	87.5 ± 1.5	96.7 ± 1.0	0.35	0.761
MCH (pg)	28.5 ± 1.2	32.2 ± 0.9	1.21	0.211
MCHC (g/dL)	29.3 ± 0.2	24.2 ± 0.9	0.71	0.1216

## 4 Discussion

Malaria and HIV co-infection in children is a condition of great public health concern as both infections have devastating effects on the health of children especially those less than 5 years old. The Overall prevalence of malaria parasitemia was 31.01 %. Our result is higher than the 24.8 % prevalence of malaria among HIV-infected children in Mutengene reported by Bate et al. (2016), 13.04 % reported in Buea Health District by Isah et al. (2020), and the 14 % malaria parasite prevalence reported in Rwanda in HIV- unexposed children (Habyarimana and Ramroop, 2020). The increased malaria burden in our study is probably due to the presence of several mosquito breeding sites that persisted all year long as well as a high prevalence of mosquito resistance to DDT and permethrin insecticides in Douala (Antonio-Nkondjio et al., 2012). Our result was however lower than the 55.0 % recorded in Limbe (Sandie et al., 2019), and the 39.4 % prevalence of malaria reported among HIV-infected children in a meta-analysis study in sub-Saharan countries (Kwenti, 2018). This discrepancy may be attributed to some state policies and strategies that have recently been implemented to fight malaria in Cameroon such as the implementation of long-lasting insecticide nets and the use of artemisinin-based combination therapy (ACT) as recommended by the World Health Organization. Also, the free distribution of ART to HIV/AIDS patients in the country as the main control measure to improve their immune status and slow down the deteriorating effect of the virus has reduced the burden of opportunistic infection.

The prevalence of anaemia in children in the study population was 25.44 % (73). This result was lower than values reported by Bate et al. (2016), and Nyesigire Ruhinda et al. (2012). This may be due to the wide-scale use of anti-malarial preventive measures that caused the decrease in the prevalence of malaria infections in these children and also the use of co-trimoxazole prescribed along with ART which has been associated with reduced anaemia in HIV-infected children (Prendergast et al., 2011). This confirms the fact that malaria is one of the major contributing factors to anaemia in children (Omarine Nlinwe and Nange, 2020).

Malaria parasite prevalence in this study was significantly higher in children <5 years when compared with those of the older age groups, and children less than 5 years were associated with higher odds of malaria parasitemia. Our findings are in agreement with that of Bate et al. (2016), and Sandie et al. (2019), where they reported a higher prevalence of malaria among HIV-infected children <5 years. Our results are probably because in malaria-endemic areas acquired immunity to malaria is known to be both exposure- and age-dependent. Hence, older children might have developed some degree of immunity as a result of repeated malaria infections. However are results is contrary to Teh et al. (2018), who reported a higher prevalence of malaria parasitaemia in children between 5 and 9 years of age. Females had a higher prevalence of malaria parasites as compared to males in this study. The higher prevalence of malaria parasites in females compared to males observed in the study could be attributed to several factors. Biological differences, such as variations in immune responses or hormonal influences, might affect susceptibility. Additionally, socioeconomic factors and differences in access to preventive measures or healthcare between genders could also contribute to the disparity (Okiring et al., 2022). Our results are in line with that of Sandie et al. (2019), and contradict what has been reported in other studies (Kimbi et al., 2013), where a higher prevalence was observed among males than females. The reason could be due fact that males are less likely to use preventive measures such as insecticide-treated bed nets or mosquito repellents compared to females (Okiring et al., 2022).

Findings from the study also revealed that in HIV-infected children, those who presented with fever had significantly higher malaria parasite prevalence than those who did not have fever and children with fever were 1.72 times more likely to have malaria. This confirms that the fever observed was likely because of the malaria parasite infection in the patient and this is similar to other studies carried out by Teh et al. (2018) and Bouyou-Akotet et al. (2014).

In line with previous findings (Jegede et al., 2017; Mirzohreh et al., 2022), children not on ART were more likely to be infected with the malaria parasites than those on ART (Kwenti, 2018). This is in line with other studies which showed that the prevalence of malaria parasites was lower in patients who were on ART than in those who were not (Kwenti, 2018). This may be because Antiretroviral therapy (ART) improves immune function in HIV-positive individuals, reducing their susceptibility to severe malaria. Healthier individuals contribute to lower malaria transmission rates. Additionally, ART programs enhance overall health infrastructure and awareness, indirectly supporting malaria control efforts. The odds of malaria infection were higher in participants on NNRTI compared to those on PI. This finding could be because PI (lopinavir/ritonavir) can inhibit malaria parasite growth and reduce malaria incidence. A higher prevalence of malaria was observed among children with CD4 cells <200cells/μL and the odds were three times higher among those children with CD4 cells <200cells/μL. Our findings are also similar to other studies in Ghana (Tagoe and Boachie, 2012) and Nigeria (Jegede et al., 2017), which reported that HIV and malaria co-infection significantly decreases CD4 count. This could be because HIV-associated immunosuppression contributes to more frequent and more severe malaria, commonly anaemia and cerebral malaria (Obeagu and Obeagu, 2024).

From our findings, Children with viral load >1000 copies /μL were three more likely to be malaria-infected. Our results might be due to the fact HIV infection can impact malaria by altering the immune response, potentially increasing susceptibility to severe malaria and complicating treatment outcomes (Mirzohreh et al., 2022; Obeagu et al., 2024). HIV-associated immunosuppression can impair the body's ability to control malaria parasite replication, leading to more frequent and severe malaria episodes. Additionally, co-infection with HIV and malaria may exacerbate the progression of both diseases, posing significant challenges for management and control strategies (Obeagu et al., 2024).

There was an association between cotrimoxazole usage and the prevalence of malaria infection, and the HIV-infected children not on cotrimoxazole were nine times more likely to suffer from malaria. This is consistent with that of (Jegede et al., 2017); Mirzohreh et al. (2022), which reported a significantly lower prevalence of HIV and malaria coinfection among respondents on cotrimoxazole. The decrease in malaria prevalence among HIV-infected children on Cotrimoxazole may be because Cotrimoxazole inhibits folate synthesis in malaria parasites by blocking dihydrofolic acid production, disrupting their metabolic processes (Mermin et al., 2004). This interference weakens the parasite's ability to replicate and survive, ultimately reducing the severity of the infection.

From our study, it was observed that the mean values of Hb, RBC, PLT, WBC and HCT were significantly lower in malaria/ HIV co-infected patients as compared with HIV-infected patients. Specifically, the incidence of anaemia, thrombocytopenia, neutropenia and leucopenia were significantly higher in malaria-parasitised subjects compared to malaria non-parasitised control. Our results are similar to that of Erhabor et al. (2006), Omarine Nlinwe and Nange (2020) and Jegede et al. (2017), which observed the incidence of pancytopenia to be significantly higher in parasitised subjects compared to non-parasitized controls. Our results are probably because HIV/AIDS, ART, and malaria can collectively impact bone marrow function, potentially leading to anaemia, leukopenia, and thrombocytopenia. HIV/AIDS itself can cause bone marrow suppression, while certain antiretroviral medications like zidovudine, indinavir and Nevirapine, and severe malaria can further exacerbate this suppression (Kwenti, 2018). Regular monitoring of blood counts is essential to detect and manage bone marrow-related complications in individuals with HIV/AIDS, especially in areas where malaria is endemic. This study also found that the mean lymphocyte count, eosinophils, basophils count, and red cell indices were similar between HIV-infected patients and malaria/HIV co-infected patients. This observation concur with the previous finding by Sandie et al. (2019), but however it is contrary to that of Jegede et al. (2017).

## 5 Conclusions

The prevalence of malaria and anaemia among HIV-infected children in our study was 31.01 % and 25.44 %, respectively. Malaria/HIV coinfection was higher among children <5 years, those not on ART and cotrimoxazole and febrile children. Children <5 years, those between 5 and 9 years, children not on ART and Cotrimoxazole, febrile children, children with viral load >1000 copies/μL, and CD4 count <2000 cells/ μL were factors associated with malaria parasitemia among HIV-infected children. Haemoglobin levels, white blood cells, neutrophils count, and platelet counts were significantly reduced among the malaria/HIV individuals compared to HIV-infected children.

## 6 Recommendations

Therefore, the related risk factors should be given appropriate attention in HIV/malaria co-infected patients and also haematological abnormalities like leucopenia, anaemia and thrombocytopenia. To curb the burden of malaria in HIV-infected persons there is a need to increase access to treatment facilities, Cotrimoxazole prophylaxis, and ART and improve malaria control methods.

## 7 Strength and limitations of the work

### 7.1 Strength of the work

• It provides valuable insights into the interplay between malaria and HIV, emphasizing the need for integrated healthcare approaches.
• The findings underscore the importance of monitoring and managing both infections to improve health outcomes in affected children.

### 7.2 Limitations of the work

• The findings may not apply to other regions with different epidemiological profiles.
• The sample size needs to be improved to have a large representation of population

## Funding

No source of funding.

## CRediT authorship contribution statement

**Ambe Fabrice Ngwa:** Writing – original draft, Validation, Supervision, Methodology, Formal analysis, Data curation, Conceptualization, Visualization, Validation, Investigation. **Ekwi Damian Nsongmayi:** Writing – review & editing, Writing – original draft, Supervision, Data curation. **Tanyi Pride Bobga:** Investigation, Data curation. **Bih Vanessa Tita:** Writing – original draft, Investigation. **Judith Ngong Nyeme:** Writing – review & editing, Writing – original draft, Visualization, Validation. **Nyanjoh Eugine Mbuh:** Writing – review & editing, Writing – original draft.

## Declaration of competing interest

The authors declare no competing interests.
